# Circadian clocks optimally adapt to sunlight for reliable synchronization

**DOI:** 10.1098/rsif.2013.1018

**Published:** 2014-03-06

**Authors:** Yoshihiko Hasegawa, Masanori Arita

**Affiliations:** 1 Department of Biophysics and Biochemistry, Graduate School of Science, The University of Tokyo, Tokyo 113-0033, Japan; 2 RIKEN Center for Sustainable Resource Science, Kanagawa 230-0045, Japan

**Keywords:** circadian clock, phase–response curve, variational method

## Abstract

Circadian oscillation provides selection advantages through synchronization to the daylight cycle. However, a reliable clock must be designed through two conflicting properties: entrainability to synchronize internal time with periodic stimuli such as sunlight, and regularity to oscillate with a precise period. These two aspects do not easily coexist, because better entrainability favours higher sensitivity which may sacrifice regularity. To investigate conditions for satisfying the two properties, we analytically calculated the optimal phase–response curve with a variational method. Our results indicate an existence of a dead zone, i.e. a time period during which input stimuli neither advance nor delay the clock. A dead zone appears only when input stimuli obey the time course of actual solar radiation, but a simple sine curve cannot yield a dead zone. Our calculation demonstrates that every circadian clock with a dead zone is optimally adapted to the daylight cycle.

## 1. Introduction

Circadian oscillators are prevalent in organisms from bacteria to humans and serve to synchronize bodies with the environmental 24 h cycle [1,2]. Although the molecular implementation of oscillation is species-specific [3–6], every circadian clocks satisfies two requirements to achieve reliable synchronization to the environment: *entrainability* to synchronize internal time with periodic stimuli and *regularity* to oscillate with a precise period. Circadian clocks are acquired through evolution independently in bacteria, fungi, plants and animals [7]. Nonetheless, entrainability and regularity constitute major characteristics conserved in all circadian clocks [6], which strongly suggest that these two properties are essential for survival. A main source of interference with regularity is discreteness of molecular species, i.e. molecular noise [8–13]. Many studies have analysed the resistance mechanisms of circadian oscillators against noise [14–17]. Regarding entrainability, circadian clocks synchronize their internal time with the environmental cycle via sunlight, and its effect depends on the wavelength or fluence, as well as on the phase of the stimulation. However, entrainability and regularity are conflicting factors, because circadian clocks with better entrainability are sensitive not only to the periodic light stimuli, but also to the molecular noise which interferes with regularity.

**Figure 1.**
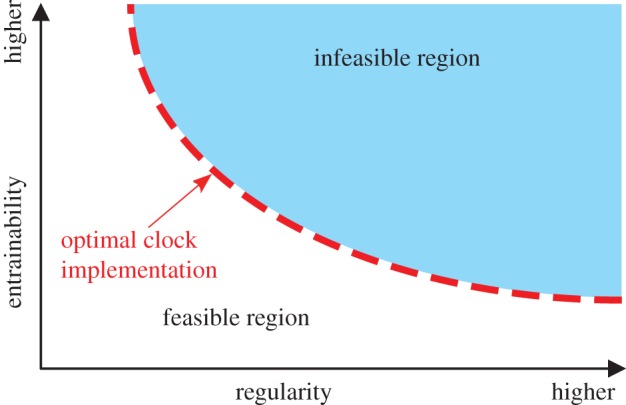
Illustrative relation between two trade-off properties: entrainability and regularity. There is an infeasible region with respect to entrainability and regularity (coloured area), inside which no clocks can be implemented. Actual circadian clocks are considered to optimally satisfy them and such optimal clocks lie on the edge between feasible and infeasible regions (thick-dashed line).

**Figure 2.**
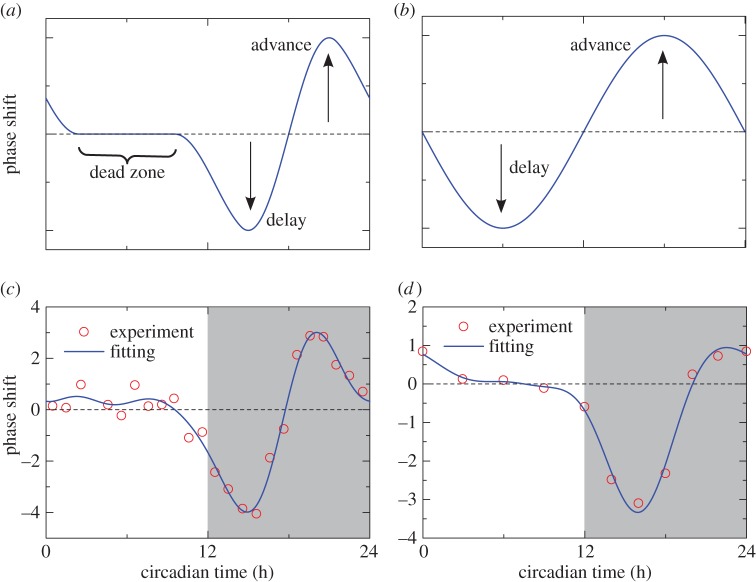
Illustrations of typical PRCs (*a*) with and (*b*) without a dead zone. Experimentally observed PRCs as a function of time in (*c*) fruitfly (*Drosophila*) [19] and (*d*) mouse (*Mus*) [20] with light pulses (circles) and their trigonometric fitting curves (solid line). Shaded and non-shaded regions indicate subjective night and day, respectively.

Because both regularity and entrainability are important adaptive values, we expect actual circadian oscillators to optimally satisfy these two factors (figure 1). Here, we investigate the optimal phase–response curve (PRC), which is both entrainable and regular, in the phase oscillator model [18] by using the Euler–Lagrange variational method. Our main finding is the inherent existence of a dead zone in the PRC: optimality is achieved only when the PRCs have a time period during which light stimuli neither advance nor delay the clock (figure 2*a*). In other words, a PRC with a dead zone (figure 2*a*) is better adapted than those without a dead zone (figure 2*b*). This result is intriguing, because a dead zone, with which oscillators tend to be unaffected by stimuli (i.e. lower entrainability), achieves better entrainability. We also tested this with two types of input stimuli: a solar radiation-type input that simulated the time course of solar radiation intensity (cf. equation (2.24) and figure 4*a*) and a simple sinusoidal input (sine curve). Surprisingly, the dead zone in the optimal PRC emerges only for the solar radiation-type input, not for the sinusoidal input. Many experimental studies reported the existence of a dead zone in various species (figure 2*c,d* show experimentally observed PRCs of fruitfly [19] and mouse [20], respectively). Our results indicate that circadian oscillators in various species have adapted to solar radiation for reliable synchronization.

## 2. Models and methods

### 2.1. Phase oscillator model

Circadian oscillators basically comprise interaction between mRNAs and proteins, whose dynamics can be modelled by differential equations. A circadian oscillator of *N* molecular species can be represented by2.1
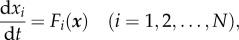
where the *N*-dimensional vector ***x*** = (*x*_1_, *x*_2_, … ,*x_N_*) denotes the concentration of molecular species (mRNAs or proteins). The effect of noise on genetic oscillators has been a subject of considerable interest, and noise-resistant mechanisms have been extensively studied [14–17,21–23]. In general, the dynamics of the *i*th molecular concentration in a circadian oscillator subjected to molecular noise is described by the following Langevin equation (Stratonovich interpretation):2.2
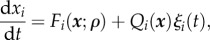
where *Q_i_*(***x***) is an arbitrary function representing the multiplicative terms of the noise, *ξ*_*i*_(*t*) is white Gaussian noise with the correlation 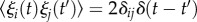 (a bracket 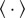 denotes expectation), and *ρ* is a model parameter.

Circadian oscillators synchronize to environmental cycles by responding to a periodic input signal (light stimuli). We let *ρ* in equation (2.2) be stimulated by the input signal: for example, *ρ* can be the degradation rate (for the sake of simplicity, we consider that the input signal affects only one parameter). We use equation (2.2) for calculating regularity and entrainability of circadian oscillators.

### 2.2. Definition of regularity

**Figure 3.**
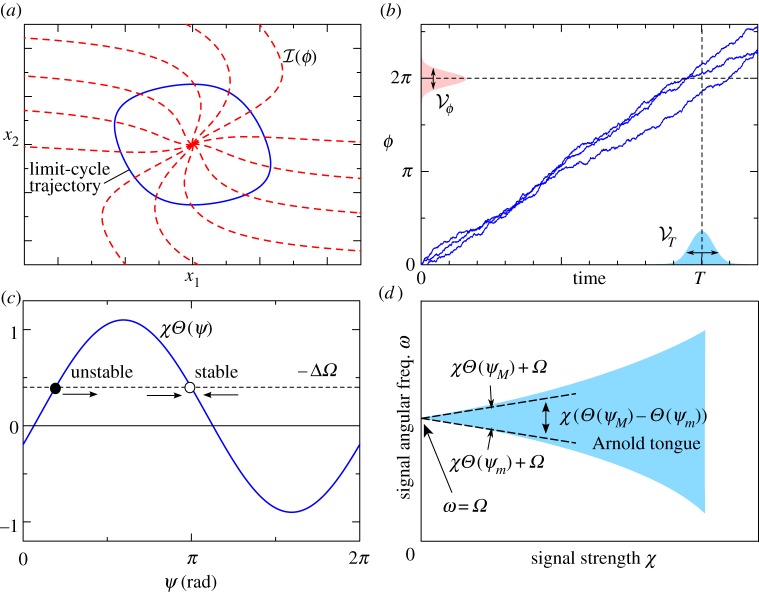
(*a*) Illustration of the isochron *ℐ*(*ϕ*), the solid and dashed lines describing a limit-cycle trajectory and its isochron drawn at intervals of *π*/6, respectively. (*b*) Relation between the phase variance 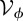 and the period variance 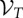 in Langevin equation (2.2) (the solid lines represent trajectories of the Langevin equation). 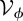 is the variance of the phase *ϕ* at time *t* = *T* and 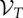 is the variance of the first passage time from 0 to 2*π*, which can be approximated by 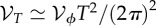. (*d*) Arnold tongue (coloured region), which shows the parameter region for synchronization to an input signal, with respect to the signal angular frequency *ω* (vertical axis) and the signal strength *χ* (horizontal axis). The dashed line is a linear approximation (equation (2.17)) of the border of the Arnold tongue when the input strength *χ* is sufficiently small.

Because the circadian oscillator of equation (2.2) is subjected to noise, its period varies cycle to cycle. We use the term regularity for the period variance of the oscillation (higher regularity corresponds to smaller period variance). Let us first consider the case without input signals (i.e. *ρ* is constant). As equation (2.1) exhibits periodic oscillation, we can naturally define the phase *ϕ* ∈ [0,2*π*) on equation (2.1) by2.3
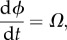
where *Ω* = 2*π*/*T* is the angular frequency of the oscillation (*T* is a period of the oscillation). The phase *ϕ* in equation (2.3) is defined only on a closed orbit of the unperturbed limit-cycle oscillation. However, we can expand the definition into the entire ***x*** = (*x*_1_, *x*_2_, … ,*x_N_*) space, where the equiphase surface is referred to as the isochron *ℐ*(*ϕ*) (figure 3*a*). By using standard stochastic phase reduction [18], equation (2.2) can be transformed into the following Langevin equation with respect to the phase variable *ϕ* (Stratonovich interpretation):2.4
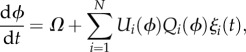
where ***U***(*ϕ*) = (*U*_1_(*ϕ*), … ,*U_N_*(*ϕ*)) is an infinitesimal PRC (iPRC) 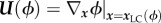, and we abbreviated *Q_i_*(***x***_LC_(*ϕ*)) as *Q_i_*(*ϕ*). iPRC *U_i_*(*ϕ*) quantifies the extent of phase advance or delay when perturbed along an *x_i_* coordinate direction at phase *ϕ*. The *N*-dimensional vector ***x***_LC_(*ϕ*) denotes a point on the limit-cycle trajectory at phase *ϕ*, where LC stands for limit cycle. The value of iPRC *U_i_*(*ϕ*) is calculated as a solution of an adjoint equation [24] or as the set of eigenvectors of a monodromy matrix in the Floquet theory [18] for arbitrary oscillators. Let *P*(*ϕ*; *t*) be the probability density function of *ϕ* at time *t*. From equation (2.4), the Fokker–Planck equation (FPE) [25] of *P*(*ϕ*; *t*) is given by2.5
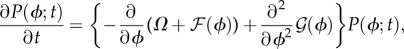
where2.6
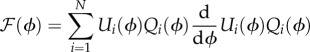
and2.7
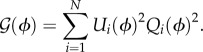
Introducing a slow variable *φ* = *ϕ* – *Ωt*, the FPE of the probability density function *Π*(*φ*; *t*) = *P*(*ϕ* = *φ* + *Ωt*; *t*) is given by2.8
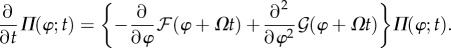

With sufficiently weak noise, *Π*(*φ*; *t*) is a slowly fluctuating function of *t*. In such cases, *ℱ*(*φ* + *Ωt*) and *𝒢*(*φ* + *Ωt*) fluctuate much faster than *Π*(*φ*; *t*), thus these two terms can be averaged for one period while keeping *Π*(*φ*; *t*) constant (phase averaging). In other words, we separate time scales between *ℱ*(*φ* + *Ωt*), *𝒢*(*φ* + *Ωt*) and *Π*(*φ*; *t*). By phase averaging, *ℱ*(*φ* + *Ωt*) vanishes because of the periodicity (use integration by parts), yielding2.9
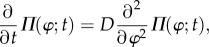
with2.10
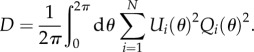
See Kuramoto [18] for further details of stochastic phase reduction and the phase-averaging procedure. From equation (2.9), because *Π*(*φ* = *ϕ*–*Ωt*; *t*)[ = *P*(*ϕ*; *t*)] obeys a simple one-dimensional diffusion equation, its solution is represented by2.11
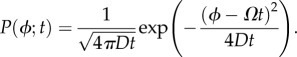


Equation (2.11) shows that the variance of the phase after one period *T* is
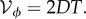


In equation (2.4), the average period corresponds to the mean first passage time with *ϕ* starting from 0 to 2*π*, and the period variance is the variance of the first passage time. Because direct calculation of the period variance is difficult, we approximate the period variance 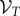 with the phase variance 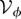, after Kori *et al.* [26]. As the phase *ϕ* crosses a threshold *ϕ* = 2*π* with gradient 2*π*/*T* without noise, there is a scaling relation 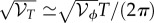 for sufficiently weak noise [26] (figure 3*b*). Consequently, the variance of the period is approximated by2.12
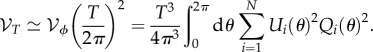


### 2.3. Definition of entrainability

The entrainment property is an important characteristic of limit-cycle oscillators and attracts attention in systems biology [27–32]. For instance, a period mismatch in coupled oscillators is known to enhance entrainability in genetic oscillators [31]. Light stimuli affect the rate constants, i.e. the parameter *ρ* in equation (2.2) is perturbed as *ρ* + d*ρ* by the input signal. Phase dynamics of equation (2.2) can be viewed as representing that of a tilted periodic potential (i.e. ratchet) subjected to noise. Because a synchronizable condition corresponds to the existence of stable points in the ratchet-like potential, the entrainability can be discussed without considering the noise. Consequently, in contrast to the calculation of regularity, in the evaluation of the entrainability, we consider a case without molecular noise (i.e. *Q_i_*(***x***) = 0 in equation (2.2)).

Let *p*(*ωt*) be an input signal with angular frequency *ω*. Considering a weak periodic input signal d*ρ* = *χp*(*ωt*), where *χ* is the signal strength (*χ* ≥ 0), and applying the phase reduction approach to equation (2.2), the time evolution of the phase variable *ϕ* is given by2.13
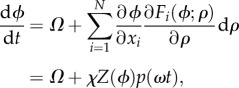
with *F_i_*(*ϕ*; *ρ*) = *F_i_*(***x***_LC_(*ϕ*); *ρ*) and2.14
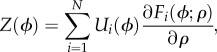
where *Z*(*ϕ*) is the PRC with respect to the parameter *ρ* and corresponds to experimentally observed PRCs. In order to distinguish *Z*(*ϕ*) from iPRC *U_i_*(*ϕ*), we will refer to *Z*(*ϕ*) as the *parametric PRC* (pPRC) [33]. Note that the definition of measured PRCs is different from pPRCs *Z*(*ϕ*) in a rigorous definition; the experimentally measured PRCs quantify the phase shift *Δϕ* caused by light stimuli, whereas pPRCs *Z*(*ϕ*) are normalized by the strength of perturbation, i.e. 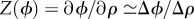. Therefore, the ranges of the measured pPRCs have limitation −*π* ≤ *Δϕ* < *π*, whereas pPRCs *Z*(*ϕ*) do not. The phase reduction can yield reliable results only when the perturbed trajectory is close to the unperturbed limit-cycle trajectory (i.e. *χ* is sufficiently small).

We next evaluate the extent of synchronization to the periodic input signal. By introducing another slow variable *ψ* = *ϕ*–*ωt* in equation (2.13), we can again apply the phase-averaging procedure, which yields2.15
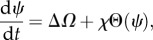
with *ΔΩ* = *Ω* – *ω* and2.16
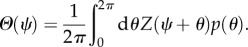
The oscillator of interest can synchronize to input signals when there is a stable solution of *ψ* in 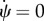 (equation (2.15)). The stable solution is an intersection point of *Θ*(*ψ*) and –*ΔΩ* with d*Θ*/d*ψ* < 0 (an empty circle in figure 3*c*). Then, a condition for the existence of a stable solution is2.17
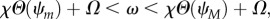
where 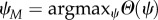 and 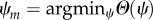.

We define entrainability, the extent of synchronization to the periodic input signal, by the width of the Arnold tongue, which is a domain with respect to *χ* (signal strength) and *ω* (signal angular frequency). The shaded region in figure 3*d* represents the Arnold tongue; with parameters *χ* and *ω* inside the Arnold tongue, the oscillator can synchronize to a periodic input signal. Because equation (2.17) constitutes a linear approximation of the Arnold tongue for sufficiently small *χ*, the width of the Arnold tongue is given by *χ*(*Θ*(*ψ*_*M*_) – *Θ*(*ψ*_*m*_)) under the linear approximation. Thus, we define the entrainability ℰ, or the extent of synchronization, as2.18
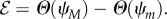


Intuitively, a circadian oscillator with better entrainability (i.e. larger ℰ) can synchronize to an input signal that has a period further from that of the oscillator. The calculation above is standard in the phase reduction approach, and further details are available in Kuramoto [18].

### 2.4. Variational method

We use the variational method to calculate the optimal PRCs which maximize the entrainability ℰ subject to constant variance 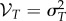 (the optimal solutions correspond to the edge in figure 1, which is described by the thick-dashed line). The constrained optimization of *U_i_*(*ϕ*) can be intuitively interpreted as maximization of weighted area (equation (2.18)), where the input being the weight, with constant area under the squared magnitude (equation (2.12)). In simple terms, the optimality is reached when the magnitude of the PRC is small during intervals when the input magnitude is small (and vice versa). In the context of neuronal oscillators, a study [34] has used the variational method to calculate the optimal PRCs for stochastic synchrony (noise-induced synchronization [35,36]).

The variational equation to be optimized is2.19
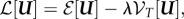
where *λ* is the Lagrange multiplier. Note that variational equation (2.19) is similar to Harada *et al*. [37], which optimizes the input signal for the maximal entrainment under constant power of the input. The variational condition *δℒ*[***U***] = 0 yields the optimal iPRC2.20
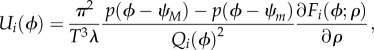
and the pPRC is calculated with equation (2.14):2.21
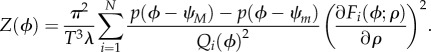
Because *ψ*_*M*_ and *ψ*_*m*_ themselves depend on *U_i_*(*ϕ*), they have to satisfy a self-consistent condition, i.e. equation (2.18) is maximal with *ψ*_*M*_ and *ψ*_*m*_. Consequently, we maximize the following function:2.22
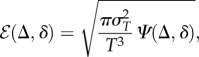
with2.23
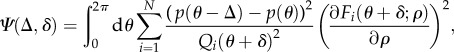
where *Δ* = *ψ*_*M*_ – *ψ*_*m*_ and *δ* = *ψ*_*m*_. The optimal iPRC can be obtained by first finding the maximum solution of *Ψ*(*Δ*, *δ*) with respect to *Δ* and *δ*, and then substituting the obtained solution *ψ*_*m*_ = *δ* and *ψ*_*M*_ = *δ* + *Δ* into equations (2.20) and (2.21).

### 2.5. Input signal of solar radiation model

**Figure 4.**
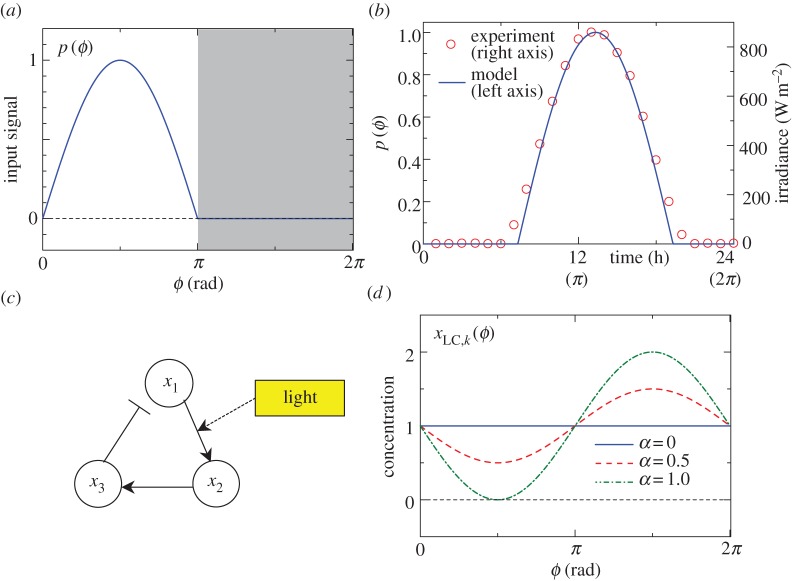
(*a*) Solar radiation input of equation (2.24), where the shaded region denotes night. (*b*) Comparison between solar radiation input (equation (2.24)) and actual observed irradiance data taken from Vick & Moss [39]. The solar radiation input (solid line) and the observed data (circle) refer to left and right axes, respectively. (*c*) Gene regulatory circuit of hypothetical circadian clock. In this example, *x*_1_ and *x*_2_ describe mRNA and protein, respectively, and *x*_3_ represses the transcription of *x*_1_. Light stimulus increases the translational efficiency. (*d*) Time course of *x*_LC,*k*_(*ϕ*) (equation (3.2)), which is a variable to be multiplied by the parameter *ρ* (equation (3.1)).

Optimal PRCs depend on input signals, as seen in equations (2.20) and (2.21). The most common synchronizer in circadian oscillators is sunlight, for which the strength is determined by 24 h-periodic solar irradiance. The solar irradiance is calculated by *I = I_0_* cos *ϑ* and *I* = 0 when the sun is above the horizon (0 ≤ *ϑ* < *π*) and below the horizon (*π* ≤ *ϑ* < 2*π*), respectively, where *ϑ* is the zenith angle and *I*_0_ is the maximum irradiance [38]. It can be approximated by2.24
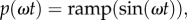
where ramp(*x*) is the ramp function defined by ramp(*x*) = *x* for *x* ≥ 0 and ramp(*x*) = 0 for *x* < 0. We call equation (2.24) the *solar radiation input*, whose plot is shown in figure 4*a* (the shaded region represents night). In order to show the validity of the solar radiation modelling, we compare equation (2.24) with observed irradiance data from Vick & Moss [39], which are shown in a dual axis plot of figure 4*b*. In figure 4*b*, equation (2.24) is plotted by the solid line (left axis) and the observed data by the dashed line (right axis). The solar radiation input of equation (2.24) is shifted horizontally, so that equation (2.24) becomes a good fit to the data. From figure 4*b*, the solar radiation input is in good agreement with the observed data, which verifies the validity of equation (2.24) as a solar radiation model.

For comparison, we also use a sinusoidal input, which is common in nonlinear sciences:2.25
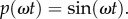


Note that *p*(*ωt*) = *B* + sin(*ωt*), where *B* is an arbitrary constant, also yields the same optimal PRCs as equation (2.25), because a constant *B* in the signal is offset in equations (2.20)–(2.23). Although a constant *B* does not play any roles in formation of the optimal PRCs, different *B* result in different Arnold tongues in general. For calculating the optimal PRCs, we use equations (2.24) and (2.25).

## 3. Results

### 3.1. Optimal phase–response curve of solar radiation input

Light stimuli generally affect the oscillatory dynamics multiplicatively, i.e. they act on the rate constants or transcriptional efficiency of the gene regulatory circuits [3,40]. We assume that the *j*th molecular species includes a parameter *ρ* as3.1
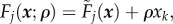
where 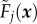 represents the terms that do not include *ρ*, and *x_k_* is the concentration of the *k*th molecular species. Here, *k*∈{1,2, … ,*N*} can take any value, regardless of *j* (both *j* ≠ *k* and *j* = *k* are allowed). For example, let figure 4*c* be a gene regulatory circuit of a hypothetical circadian clock, where symbols → and ⊣ represent positive and negative regulations and *x_i_* are molecular species (see Novák & Tyson [41] for typical motifs of biochemical oscillators). Suppose *x*_1_ and *x*_2_ are mRNA and corresponding protein, respectively, and light stimuli increase the translational efficiency. In this case, the dynamics of light entrainment can be described by equation (3.1) with *j* = 2, *k* = 1 and *ρ* being the translation rate. In equation (3.1), although we can also consider an alternative case 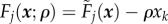 (a negative sign), the optimal pPRCs remain unchanged under the inversion which is seen from equations (2.21) and (2.23). Consequently, we consider only the positive case to calculate the optimal PRCs (i.e. equation (3.1)). However, note that relations between iPRCs and pPRCs are affected by the inversion of the sign, and the difference matters when considering biological feasibility.

When using phase reduction, the dynamics of the limit cycle are considered on the unperturbed limit-cycle trajectories ***x***_LC_, and hence the points on the limit cycle can be uniquely determined by the phase *ϕ*. Consequently, under the phase reduction, *x_k_* is replaced by *x*_LC,*k*_(*ϕ*) in equation (3.1), where *x*_LC,*k*_(*ϕ*) is the *k*th coordinate of ***x***_LC_ (i.e. 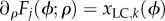 in equation (2.20)). Here, 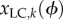 corresponds to the time course of the concentration of the *k*th molecular species. Because *x*_LC,*k*_(*ϕ*) constitutes a core clock component and is generally a smooth 2*π*-periodic function, we approximate it with a sinusoidal function:3.2
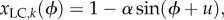
where *u* is the initial phase and *α* denotes the amplitude of the oscillation (figure 4*d*). To ensure 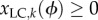, we set 0 ≤ *α* ≤ 1, and *α* = 0 recovers the additive case. Because the initial phase *u* does not play any role (*u* is offset by *δ* in equation (2.23)) when the white Gaussian noise is additive (i.e. *Q_i_*(***x***) ∝ 1), we also set *u* = 0. The parametric approximation of equation (3.2) enables an almost closed form for the overall calculations. Although we assumed in equation (3.1) that effects of *ρ* only depend on *x_k_*, we can generalize equation (3.1) to 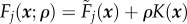, where *K*(***x***) is a nonlinear function and is assumed to be well approximated by 1 – *α*sin(*ϕ* + *u*). By this generalization, our theory can be applied to other possible light entrainment mechanisms such as the intercellular coupling [42]. Our model needs only details about molecular species which have light input entry points but not about a whole molecular network. However, this advantage, in turn, means that we cannot specify iPRCs *U_i_*(*ϕ*) of molecular species not having light input entry points. Consequently, for a noise term *Q_i_*(***x***), we assume that the white Gaussian noise is additive and is present only in the *j*th coordinate equation (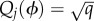, where *q* is the noise intensity and *Q_i_*(*ϕ*) = 0 for *i* ≠ *j*).

**Figure 5.**
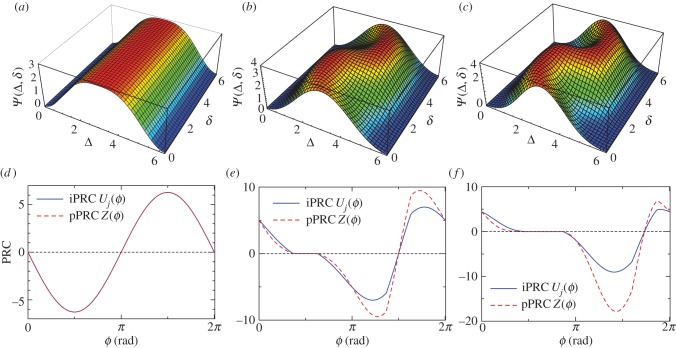
(*a*–*c*) Landscape of *Ψ*(*Δ*, *δ*) as a function of *Δ* and *δ* with solar radiation input (equation (2.24)) for (*a*) *α* = 0, (*b*) *α* = 0.5 and (*c*) *α* = 1.0, where the maximum points are parameters for the optimal PRC. (*d*–*f*) Optimal PRCs with solar radiation input: (*d*) *α* = 0, (*e*) *α* = 0.5 and (*f*) *α* = 1.0. In (*d*–*f*), the solid and dashed lines denote iPRCs *U_j_*(*ϕ*) and pPRCs *Z*(*ϕ*), respectively (in (*d*), solid and dashed lines are indistinguishable). The maximal parameters (*Δ*,*δ*) for (*d*–*f*) are (*d*) (*π*, 0), (*e*) (2.31, 1.99) and (*f*) (2.30, 2.72). In (*d*), a parallel shift of the PRC is also optimal (*δ* can be an arbitrary value). In (*e*), symmetric PRCs with respect to the horizontal axis are also optimal. In (*f*), symmetric PRCs with respect to the horizontal axis or *ϕ* = 3*π*/2 are also optimal (see the text). The pPRCs correspond to experimentally observed PRCs.

Figure 5*a–c* shows the landscape of *Ψ*(*Δ*,*δ*) as functions of *Δ* and *δ*, and figure 5*d–f* expresses the optimal iPRCs *U_j_*(*ϕ*) and pPRCs *Z*(*ϕ*) for the solar radiation input (an explicit expression of *Ψ*(*Δ*, *δ*) is given in appendix A). The optimal PRC shape does not depend on the model parameters such as the period *T*, its variance 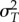, or noise intensity *q*. These three parameters only act on the magnitude of the PRCs (i.e. the vertical scaling of the PRCs). Consequently, we normalized *T* = 1, 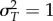, and *q* = 1, as shown in figure 5. As the optimal PRCs depend on *α*, *Ψ*(*Δ*, *δ*) is plotted for three cases: *α* = 0, (figure 5*a*), *α* = 0.5 (figure 5*b*) and *α* = 1.0 (figure 5*c*), where the maximal points (*Δ*, *δ*) yield the optimal PRCs using equations (2.20) and (2.21). The maximal parameters *Δ* and *δ* are calculated numerically. Figure 5*d–f* describes the optimal iPRCs (solid line) and pPRCs (dashed line) for *α* = 0, 0.5 and 1.0, respectively. When *α* = 0, i.e. the input signal is additive, *Ψ*(*Δ*,*δ*) achieves a maximum for *Δ* = *π* and arbitrary *δ*, yielding sinusoidal PRCs as the optimal solution (figure 5*d*). Although the input signal *p*(*ϕ*) is not sinusoidal, the optimal PRCs obtained using the variational method become sinusoidal. In other words, considering optimality, resonator-type oscillators have an advantage over integrator-type oscillators. For *α* > 0, the input signal *p*(*ϕ*) depends on the concentration of the *k*th molecular species. From figure 5*b*, the optimal parameters for *α* = 0.5 are (*Δ*, *δ*) = (2.31, 1.99) and (3.98, 4.30), which are different from *Δ* = *π* (these two sets yield symmetric PRCs with respect to the horizontal axis). Figure 5*e* shows the optimal iPRCs *U_j_*(*ϕ*) and pPRCs *Z*(*ϕ*) for *α* = 0.5. Interestingly, the optimal iPRCs and pPRCs for *α* = 0.5 have a dead zone (region of 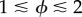 in figure 5*e*) in which the input signal neither advances nor delays the clock. From equations (2.20)–(2.21) and the solar radiation input of equation (2.24), the optimal PRCs inevitably include a dead zone if the optimal *Δ* is not *π*. For *α* = 1.0, there are four sets of parameters (*Δ*, *δ*) that give optimal PRCs: (2.30, 2.72), (2.30, 1.26), (3.98, 3.56) and (3.98, 5.02) (PRCs with these four sets are symmetric with each other with respect to the horizontal axis or *ϕ* = 3*π*/2). Consequently, the optimal PRCs shown in figure 5*f* have a dead zone as in the case of *α* = 0.5.

### 3.2. Dead zone length

**Figure 6.**
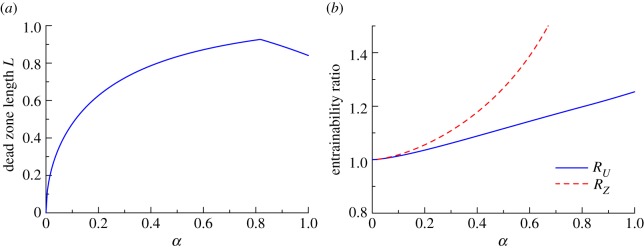
(*a*) *α*-Dependence of the dead zone length *L*. (*b*) *α*-Dependence of the entrainability ratios *R_U_* (solid line) and *R_Z_* (dashed line; equation (3.6)). *R_U_* and *R_Z_* are the ratios of the entrainability of the optimal PRC to that of the sinusoidal iPRC (equation (3.4)) and the pPRC (equation (3.5)), respectively.

From the results discussed above, the optimal PRCs have a dead zone when *α* > 0. We next studied the length of the dead zone as a function of *α* (figure 6*a*) and improvements in the entrainability induced by the dead zone (figure 6*b*) for the solar radiation input. Because the dead zone, which is a null interval in PRCs, emerges when the optimal parameter is *Δ* ≠ *π*, we can naturally define its length as3.3
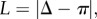
where *Δ* is the maximum value of *Ψ*(*Δ*, *δ*). As seen in figure 6*a*, a dead zone clearly exists when *α* > 0, and the length increases with increasing *α* for *α* < 0.8. Even for *α* = 0.1, when the oscillation amplitude of *x*_LC,*k*_(*ϕ*) (the concentration of a molecular species modulated by the light-sensitive parameter *ρ*; cf. figure 4*d*) is very small, we observe a dead zone with a length of *L* = 0.475, which corresponds to about 3 h within 24 h, indicating the universality of having a dead zone in order to attain optimality. The improvement in the entrainability that is induced by a dead zone is calculated by comparing the entrainability of the optimal PRCs with that of typical sinusoidal PRCs. We consider sinusoidal functions for both the iPRC *U_j_*(*ϕ*) and pPRC *Z*(*ϕ*) by setting3.4
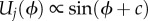
and3.5
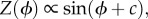
where *c* is the parameter to be optimized so that entrainability is maximized for each *α* (see appendix B for the explicit expressions). Equations (3.4) and (3.5) are scaled, so that they satisfy the constraints on the period variance (equation (2.12)). We calculated the ratios3.6
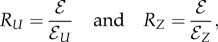
where 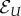 and 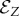 represent the entrainabilities for the cases of the sinusoidal iPRC and pPRC, respectively, calculated for the solar radiation input. For the sinusoidal iPRC of equation (3.4), the entrainability is calculated with pPRC via equation (2.14). *R_U_* and *R_Z_* quantify the improvement rate of the optimal PRCs over the sinusoidal iPRC (*R_U_*) and pPRC (*R_Z_*). In figure 6*b*, the solid and dashed lines show *R_U_* and *R_Z_*, respectively, as a function of *α*. Both ratios monotonically increase as *α* increases, which shows that the optimal PRC with a dead zone exhibits better entrainability when the oscillation of *x*_LC,*k*_(*ϕ*) has a larger amplitude. When the concentration of *x*_LC,*k*_(*ϕ*) is low, the effects of the input signal on the circadian oscillators are smaller. This is because pPRC *Z*(*ϕ*), which quantifies the extent of the phase shift owing to the stimulation of the parameter, depends on the concentration *x*_LC,*k*_(*ϕ*) (see equation (2.14)). However, even within the range *ϕ* where *x*_LC,*k*_(*ϕ*) has smaller values, the iPRC *U_j_*(*ϕ*) contributes to an increase in the variance of the period, regardless of the concentration. From this, we see that having an iPRC with a smaller magnitude when the concentration of *x*_LC,*k*_(*ϕ*) is smaller results in a smaller variance, which results in a larger entrainability for a constant variance of the period. Although this qualitatively explains the benefit of a dead zone, for some input values, the optimal PRCs may not contain a dead zone for any value of *α*. This will be shown in the following.

### 3.3. Optimal phase–response curve of sinusoidal input

**Figure 7.**
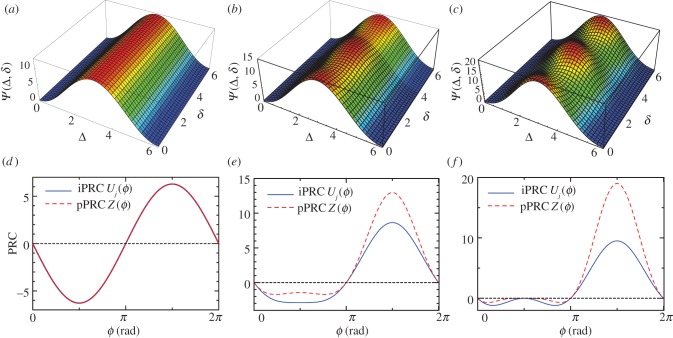
(*a*–*c*) Landscape of *Ψ*(*Δ*, *δ*) as functions of *Δ* and *δ* with sinusoidal input for (*a*) *α* = 0, (*b*) *α* = 0.5 and (*c*) *α* = 1.0, where the maximum points are parameters for the optimal PRC. (*d*–*f*) Optimal PRCs with sinusoidal input (equation (2.25)): (*d*) *α* = 0, (*e*) *α* = 0.5 and (*f*) *α* = 1.0, where the solid and dashed lines denote iPRCs *U_j_*(*ϕ*) and pPRCs *Z*(*ϕ*). The maximal parameter (*Δ*, *δ*) is (*π*, 0) in all cases. In (*d*), a parallel shift of the PRC is also optimal (*δ* can be an arbitrary value). PRCs that are symmetric with respect to the horizontal axis are also optimal. The pPRCs correspond to experimentally observed PRCs.

Because the optimal PRCs depend on input signals (equations (2.20) and (2.21)), we next consider a typical periodic input signal, a sinusoidal function (equation (2.25)). In this case, *Ψ*(*Δ*, *δ*) is calculated in a closed form (an explicit expression of *Ψ*(*Δ*, *δ*) is given in appendix A), which is plotted as functions of *Δ* and *δ* in figure 7*a–c* for three cases: *α* = 0 (figure 7*a*), *α* = 0.5 (figure 7*b*) and *α* = 1.0 (figure 7*c*). As can been seen from figure 7*a–c*, *Ψ*(*Δ*, *δ*) yields the maximal value for (*Δ*, *δ*) = (*π*, *n*π**) for 0 < *α* ≤ 1, where *n* is an integer and when *α* = 0, *δ* can take any value. Figure 7*d–f* expresses the optimal iPRCs *U_j_*(*ϕ*) and pPRCs *Z*(*ϕ*) for the sinusoidal input. For *α* = 0, the optimal PRC is sinusoidal (figure 7*d*) and for *α* = 0.5, the optimal PRC is still close to a sinusoidal function (figure 7*e*). When increasing *α* to *α* = 1.0, the PRC diverges from the sinusoidal function and exhibits almost positive values (figure 7*f*). We see that the optimal PRCs owing to equations (2.20) and (2.21) do not exhibit a dead zone for any *α*-values (figure 7*d–f*) when the input signal is a simple sinusoidal function.

## 4. Discussion

The existence of a dead zone optimizes both entrainability and regularity. It is rather obvious that optimization of regularity alone leads to a dead zone [43], because null response means no effect by any kind of fluctuations. Our result instead shows that optimality of both entrainability and regularity, which are in a trade-off relationship, is uniquely achieved by a dead zone. Our finding is fairly general, because a dead zone always exists in an optimal PRC unless *α* = 0 (additive stimulation). Along with the fact that *T*, σ*_T_* and *q* affect only the scaling of the optimal PRCs, when the input signal affects the dynamics multiplicatively (i.e. *α* > 0), the existence of a dead zone always provides a synchronization advantage. This is supported by many experimental studies of various species that report the existence of a dead zone in the PRC [1] (cf. figure 2*c,d*). Our general result suggests that circadian oscillators have fully adapted to solar radiation to improve synchronization. Indeed, many experimental findings imply that circadian oscillators have adapted to actual solar radiation [44]: for various animals, light–dark (LD) cycles that include a twilight period result in better entrainability than do abrupt LD cycles (on–off protocols) [44]. In this regard, another interesting problem is optimal entrainment [37] of circadian clocks by light stimuli. As two different input signals, the solar radiation and sinusoidal inputs, yield the same optimal PRCs for *α* = 0, optimal inputs and optimal PRCs do not have one-to-one correspondence. Thus, the optimal inputs are not trivial and this problem should be pursued in our future studies.

The solar radiation input plays an essential role, because it yields a dead zone in the optimal PRC, whereas a sinusoidal signal does not (figure 7). In other words, oscillators that are entrained by stimuli other than solar radiation may not exhibit a dead zone in their PRCs. This is indeed found in mammals. Mammals possess a master clock in their suprachiasmatic nucleus (SCN), which receives light stimuli via retinal photoreceptors, and peripheral clocks in body cells [45]. The peripheral oscillators are entrained by several stimuli such as feeding and signals from the SCN through chemical pathways (e.g. hormones) [45,46]. By injection experiments of a hormone, Balsalobre *et al.* [47] reported that the PRCs of the peripheral oscillators in the liver do not have a dead zone.

Our result also agrees with other experimental observations. Our theory implies that a dead zone should be located where the concentration *x*_LC,*k*_(*ϕ*) is low (0 ≤ *ϕ* ≤ *π* in figure 4*d*), and that to achieve optimality, the concentration of *x*_LC,*k*_(*ϕ*) should be maximal in the region where the PRCs exhibit a large phase shift. In *Drosophila*, the *timeless* (*tim*) gene is regarded as the molecular implementation of *x*_LC,*k*_(*ϕ*). It is experimentally known that light enhances the degradation of the gene product (the TIM protein) [48,49], and the TIM protein peaks during the late evening. Figure 2*c* shows observations of the PRC of *Drosophila* against light pulses as a function time from Hall & Rosbash [19]; circles describe the experimental data, and the solid line expresses a trigonometric curve fitting (fourth order). Because the centre of the part of the PRC that can be phase shifted approximately corresponds to the peak of the concentration, as denoted above, when estimated from the PRC alone, the concentration peak of the TIM protein should occur at about 18 h. This time is also close to the experimental evidence (i.e. late evening). Therefore, our theory can be used to hypothesize further molecular behaviour affected by light stimuli.

In summary, we have constructed a model that regards circadian oscillators as a global optimization of entrainability and regularity. We have shown that our model is consistent with much experimental evidence as mentioned above. The extension and improvement of our method are possible and they are left as an area of future study.

## Appendix A. Explicit expression of *Ψ*(*Δ*, *δ*)

**A.1. Solar radiation input case**

For the solar radiation input case (equation (2.24)), *Ψ*(*Δ*, *δ*) is given by

A1

with

where . We show equation (A 1) as functions of *Δ* and *δ* in figure 5*d–f*.

**A.2. Sinusoidal input case**

For the sinusoidal input case (equation (2.25)), *Ψ*(*Δ*, *δ*) is given by

A2

We plot equation (A 2) as functions of *Δ* and *δ* in figure 7*d–f*.

## Appendix B. Explicit expression of sinusoidal phase–response curves

**B.1. Sinusoidal infinitesimal phase–response curve**

An explicit expression for the sinusoidal iPRC (equation (3.4)) is

B1

which yields the period variance of . Then, the corresponding pPRC is given by

B2

where we used equation (2.14).

**B.2. Sinusoidal parametric phase–response curve**

For the pPRC *Z*(*ϕ*) to be a sinusoidal function, the iPRC *U_j_*(*ϕ*) must be

B3

where we used equation (2.14). An explicit expression of equation (B 3) is

B4

where *𝒩*(*c*) is a normalizing term

Equation (B 4) is normalized, so that the period variance becomes . Using equation (2.14), the corresponding pPRC is a sinusoidal function:

B5

which is an explicit expression of the sinusoidal pPRC (equation (3.5)).
